# Efficacy and safety of ultrasound-guided microwave ablation versus surgical resection for Bethesda category IV thyroid nodules: A retrospective comparative study

**DOI:** 10.3389/fendo.2022.924993

**Published:** 2022-09-23

**Authors:** Jingjing Yang, Ya Zhang, Xingjia Li, Yueting Zhao, Xue Han, Guofang Chen, Xiaoqiu Chu, Ruiping Li, Jianhua Wang, Fei Huang, Chao Liu, Shuhang Xu

**Affiliations:** ^1^ Endocrine and Diabetes Center, Affiliated Hospital of Integrated Traditional Chinese and Western Medicine, Nanjing University of Chinese Medicine, Jiangsu Province Academy of Traditional Chinese Medicine, Nanjing, China; ^2^ Key Laboratory of Traditional Chinese Medicine (TCM) Syndrome and Treatment of Yingbing (Thyroid Disease) of State Administration of Traditional Chinese Medicine, Jiangsu Province Academy of Traditional Chinese Medicine, Nanjing, China; ^3^ Department of Pathology, Affiliated Hospital of Integrated Traditional Chinese and Western Medicine, Nanjing University of Chinese Medicine, Nanjing, China; ^4^ Department of General Surgery, Affiliated Hospital of Integrated Traditional Chinese and Western Medicine, Nanjing University of Chinese Medicine, Nanjing, China; ^5^ Department of Endocrinology, Suzhou Traditional Chinese Medicine (TCM) Hospital Affiliated to Nanjing University of Chinese Medicine, Nanjing, China

**Keywords:** thyroid nodule, the Bethesda system for reporting thyroid cytology, microwave ablation, thyroidectomy, follicular neoplasm (FN)/suspicious for follicular neoplasm (SFN)

## Abstract

**Objective:**

The objective of this study was to assess the efficacy and safety of ultrasound-guided microwave ablation (MWA) for Bethesda IV thyroid nodules and to compare the outcomes, complications, and costs of MWA and thyroidectomy.

**Methods:**

A total of 130 patients with Bethesda IV nodules were retrospectively reviewed, involving 46 in the MWA group and 84 in the surgery group. The local institutional review board approved this study. Patients in the MWA group were followed up at 1, 3, 6, and 12 months after the intervention. Postoperative complications, treatment time, and cost in the two groups were compared.

**Results:**

Among 84 patients with 85 Bethesda IV nodules in the surgery group, postoperative pathology was benign lesions, borderline tumors, papillary thyroid carcinoma, follicular variant papillary thyroid carcinoma, follicular thyroid carcinoma, and medullary carcinoma in 44, 4, 27, 6, 3, and 1 cases, respectively. Malignant thyroid nodules were more prone to solid echostructure (86.11% *vs.* 72.72%), hypoechogenicity (55.56% *vs.* 13.63%), and irregular margin (47.22% *vs.* 13.63%) than benign lesions. The nodule volume reduction rate of patients at 12 months after MWA was 85.01% ± 10.86%. Recurrence and lymphatic and distant metastases were not reported during the follow-up period. The incidence of complications, treatment time, hospitalization time, incision length, and cost were significantly lower in the MWA group than in the surgery group (all *p* < 0.001).

**Conclusions:**

MWA significantly reduces the volume of Bethesda IV nodules with high safety and is recommended for those with surgical contraindications or those who refuse surgical resection. Patients with suspicious ultrasound features for malignancy should be actively treated with surgery.

## Introduction

Fine-needle aspiration cytology (FNAC) is a major tool to determine whether a thyroid nodule is cancerous. The Bethesda System for Reporting Thyroid Cytopathology (TBSRTC) has standardized the diagnostic terms and morphologic criteria in thyroid cytology, thus facilitating communication among cytopathologists, clinicians, and investigators. Since the first edition was proposed in 2007, TBSRTC has been recommended by the American Thyroid Association (ATA) as the guideline for the management of thyroid nodules in adults and children (1, 2).

Recent research has upgraded this system; the second edition of TBSRTC was published in 2017. Thyroid nodules are classified into six categories (I–I category) in TBSRTC, and Bethesda IV accounts for 2%–25% in all thyroid fine-needle aspiration samples (3). As an indeterminate cytology type including follicular neoplasm (FN) or suspicious for a follicular neoplasm (SFN), Bethesda IV is estimated to have a 10%–40% risk of malignancy (1–3). Ren et al. (4) reported that among 300 thyroid nodules confirmed by pathological examination, the malignant rate of Bethesda IV is 25%. Bethesda IV nodules are characterized as highly crowded conditions of cells and/or the presence of follicular cells. As a type of follicular epithelial tumor, FN lacks papillary-like nuclear features, with a spectrum covering follicular thyroid adenoma (FA), follicular thyroid carcinoma (FTC), and follicular variant papillary thyroid carcinoma (FVPTC) (5). It is still a challenge to differentiate benign from malignant FN by FNAC, because of the similar or even overlapping morphological features of different subtypes of FN (6). Postoperative histopathology is the gold standard for diagnosing FTC, based on the presence of vascular or capsular invasion (7). Therefore, diagnostic thyroidectomy is considered a standard treatment for FN (1). About 80% of FN samples are postoperatively diagnosed as benign (8). In addition, conservative treatment is preferred for patients with FN but not suitable for surgery, because of concerns about surgical trauma and relevant complications, like hypothyroidism and hoarseness (9).

Ultrasound-guided thermal ablation has been used to treat benign thyroid diseases, papillary thyroid carcinoma (PTC), and metastatic neck tumors (10–13). However, the efficacy of microwave ablation (MWA), a typical technology in thermal ablation, is still debatable in treating Bethesda IV nodules. A literature review has just collected scant data supporting the efficacy and safety of MWA for FN. In the present study, we retrospectively analyzed the clinical data of 46 and 84 cases of Bethesda IV nodules that underwent intervention by ultrasound-guided MWA and thyroid lobectomy/total thyroidectomy, respectively. Through a 12-month follow-up, the outcomes, complications, and costs of the two methods were recorded and compared.

## Materials and methods

### Patients

This retrospective study was approved by the ethics committee of the Jiangsu Province Academy of Traditional Chinese Medicine (MS2018074). Written informed consent was obtained from all patients prior to ultrasound-guided FNAC, MWA, and surgery. The informed consent emphasized that MWA cannot fully avoid the potential risks of recurrence and distant metastasis.

**Figure 1:**
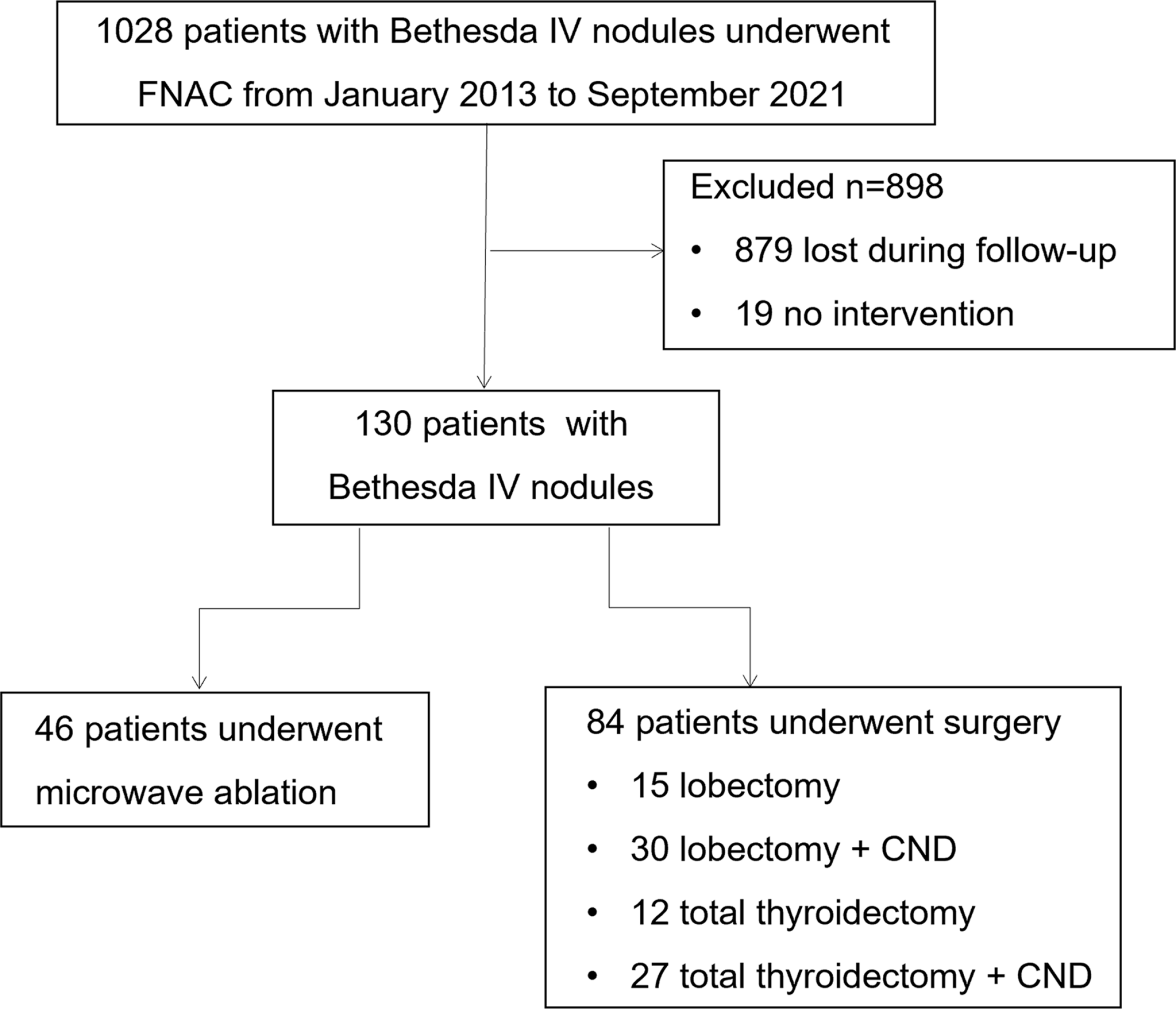
Flowchart summarizing the patient inclusion process. FNAC, fine-needle aspiration cytology; CND, central lymph node dissection.

A total of 130 patients with Bethesda IV nodules diagnosed by FNAC and treated with MWA or thyroid lobectomy/total thyroidectomy in the Affiliated Hospital of Integrated Traditional Chinese and Western Medicine of Nanjing University of Chinese Medicine from January 2013 to September 2021 were retrospectively reviewed, involving 46 in the MWA group and 84 in the surgery group (**Figure 1**). The inclusion criteria were as follows: 1) men or women aged 18–75 years, 2) patients examined by color Doppler ultrasound in the Affiliated Hospital of Integrated Traditional Chinese and Western Medicine of Nanjing University of Chinese Medicine, 3) Bethesda category IV thyroid nodules diagnosed by FNAC, 4) no history of neck surgery and malignant tumors, 5) absence of vocal cord abnormalities and recurrent laryngeal nerve damage confirmed by laryngoscopy, and 6) willingness to undergo MWA or surgical resection and provided written informed consent. The exclusion criteria were as follows: 1) combined with non-thyroid diseases like parathyroid adenoma, tuberculosis, and other tumors; 2) history of surgery for malignant thyroid tumors like PTC, medullary thyroid cancer (MTC), and FTC; 3) allergic reactions to local anesthetics, analgesics, and hemostatic drugs; 4) severe bleeding diathesis or platelet count <50 × 10^9^/L; 5) coagulopathy (prothrombin time (PT) >25 s and prothrombin time activity <40%) or long-term use of aspirin, warfarin, and other anticoagulants that had not been withdrawn within 1 week prior to the surgery; 6) systemic infection, high fever (>38.5°C), or white blood cell count >10 × 10^9^/L or <4 × 10^9^/L; 7) consciousness disorder or unable to comply with the treatment; 8) severe organ dysfunction; and 9) other conditions determined by investigators.

### Preoperative assessment

All patients were examined by preoperative ultrasound (Hi ViSIOn Preirus, HITACHI, Tokyo, Japan) to assess the anteroposterior, transverse, and longitudinal diameters, volume, location, composition, echogenicity, shape, margin, and calcifications of the thyroid nodule. The thyroid nodule volume was calculated as V = πabc/6, where “a” is the largest diameter and “b” and “c” are the two perpendicular diameters.

### Ablation procedure

The MWA procedures were performed by one physician with broad experience in thyroid interventional ultrasound. A 2,450-MHz MWA system consisting of a KY-2000 microwave generator (Kangyou Applied Research Institute, Nanjing, China) and a disposable sterile MWA needle was used. The microwave generator producing 25–30 W of output power was connected with a 16G water-cooled microwave antenna through a low-loss microwave coaxial cable. The heating point was 3 mm away from the tip of the microwave antenna. The Siemens Acuson S2000 ultrasound machine with an 18L6 probe at 6–18 MHz was used in the MWA system.

MWA was performed in an outpatient operating room. The patient was in the supine position with the neck fully exposed and monitored by electrocardiogram and pulse oximetry. The localization and extent of ablation areas, as well as the ablation path, were predetermined through ultrasound examinations. After disinfection with 0.2% iodine and overlaying with sterile sheets, ultrasound-guided local infiltration anesthesia was performed using 1% lidocaine at the lesion site. Through the hydro-dissection technique, normal saline was continuously injected in the space between the thyroid capsule and trachea, esophagus, laryngeal nerve, and parathyroid gland to safeguard vital structures of the neck. Under the guidance of ultrasound, the ablation antenna was inserted into the lesion with the previously determined path, and MWA producing 25–30 W of output power was started. An area of 0.5–1 cm beyond the edge of the tumor was ablated to prevent tumor residues and recurrence under adequate and continuous hydro-dissection. An immediate intraoperative contrast-enhanced ultrasound (CEUS) was performed to assess the therapeutic effect. The ablation antenna was gently pulled out, and the ablation area was ice-packed for 4 h to prevent bleeding and reduce swelling. Vital signs, phonation, and swallowing were closely monitored during MWA. After ablation, this area was compressed with an ice pack. Skin burns, hoarseness, damage to the trachea and esophagus, and other discomforts were recorded after MWA. The patient returned to the ward after close monitoring for 0.5 h.

### Surgical procedures

Thyroid lobectomy or total thyroidectomy was performed by experienced physicians. Briefly, the patient was placed in the supine position with the shoulders padded by a cushion. After general anesthesia and endotracheal intubation, a collar incision at one fingerbreadth above the sternal notch was created to sequentially expose the skin, subcutaneous tissues, and platysma. Flaps between the deep cervical fascia and loose connective tissues of the platysma were separated from the edge of the thyroid cartilage to the sternal notch. The linea alba cervicalis was divided vertically until the capsule of the thyroid was exposed. After blood vessels around the thyroid were ligated and the recurrent laryngeal nerve was exposed, thyroid lobectomy or total thyroidectomy was performed with *in situ* preservation of parathyroid glands. Tissue samples were subjected to pathological examinations. Negative pressure drainage was performed, followed by suturing and assessment of neurological function and hemorrhage.

### Post-ablation evaluation and follow-up

The extent of ablation was immediately assessed after MWA by thyroid ultrasound and contrast-enhanced ultrasound. Complete ablation of the thyroid nodule was ensured. Patients were followed up at 1, 3, 6, and 12 months after the intervention. The therapeutic effect was evaluated by ultrasound, and volume of ablation, recurrence, and lymph node metastasis was detected. The percentage reduction of the ablation area in volume was calculated as follows: volume reduction ratios (VRR) = (initial volume − final volume) × 100%/initial volume.

### Statistical analysis

Statistical analysis was performed using SPSS 23.0. Continuous variables were expressed as mean ± standard deviation ( ± s). For normally distributed measurement data, between-group comparison was performed through the independent sample *t*-test and their intragroup comparison through the paired sample *t*-test. For measurement data that did not conform to normality, between-group comparison was performed by non-parametric tests on two independent samples, and their intragroup comparison was performed by two sample non-parametric tests. Binary variables were compared by Pearson’s chi-square test. The maximum volume and VRR at each time point of follow-up were compared by the Wilcoxon rank sum test. *p* < 0.05 is considered statistically significant.

## Results

### Baseline characteristics

A total of 130 patients with 132 Bethesda category IV thyroid nodules were retrospectively reviewed, involving 24 (18.46%) men and 106 (81.53%) women. They were assigned to the MWA group (n = 46, 47 thyroid nodules) and the surgery group (n = 84, 85 thyroid nodules). There were 11 (23.40%) men and 35 (74.46%) women with a mean age of 20–67 (45.96 ± 11.66) years in the MWA group and 13 (15.48%) men and 71 (84.52%) women with a mean age of 21–73 (46.10 ± 12.11) years in the surgery group. No significant differences were detected in the age and sex between groups (both *p* > 0.05). The maximum diameter (3.12 ± 0.86 *vs.* 2.32 ± 1.54 cm) and volume of thyroid nodules (7.54 ± 76.64 *vs.* 5.44 ± 8.41 ml) were significantly larger in the MWA group than in the surgery group (both *p* < 0.05, **Table 1**).

**Table 1:** Baseline characteristics of patients with Bethesda category IV thyroid nodules (n = 132).

	MWA group	Surgery group
Thyroid nodules (n, %)	47 (35.60)	85 (64.08)
Case number (n, %)	46 (35.38)	84 (65.94)
Age (years)	46.30 ± 11.54	46.10 ± 12.11
Sex		
Male (%)	11 (23.91%)	13 (15.48%)
Female (%)	35 (74.47%)	71 (84.52%)
The volume of thyroid nodule (ml)	7.47 ± 6.68*	5.44 ± 8.41
The maximum diameter of thyroid nodule (cm)	3.12 ± 0.86*	2.32 ± 1.54

MWA, microwave ablation.

*p < 0.05 vs. surgery group.

### Preoperative ultrasound characteristics

**Figure 2:**
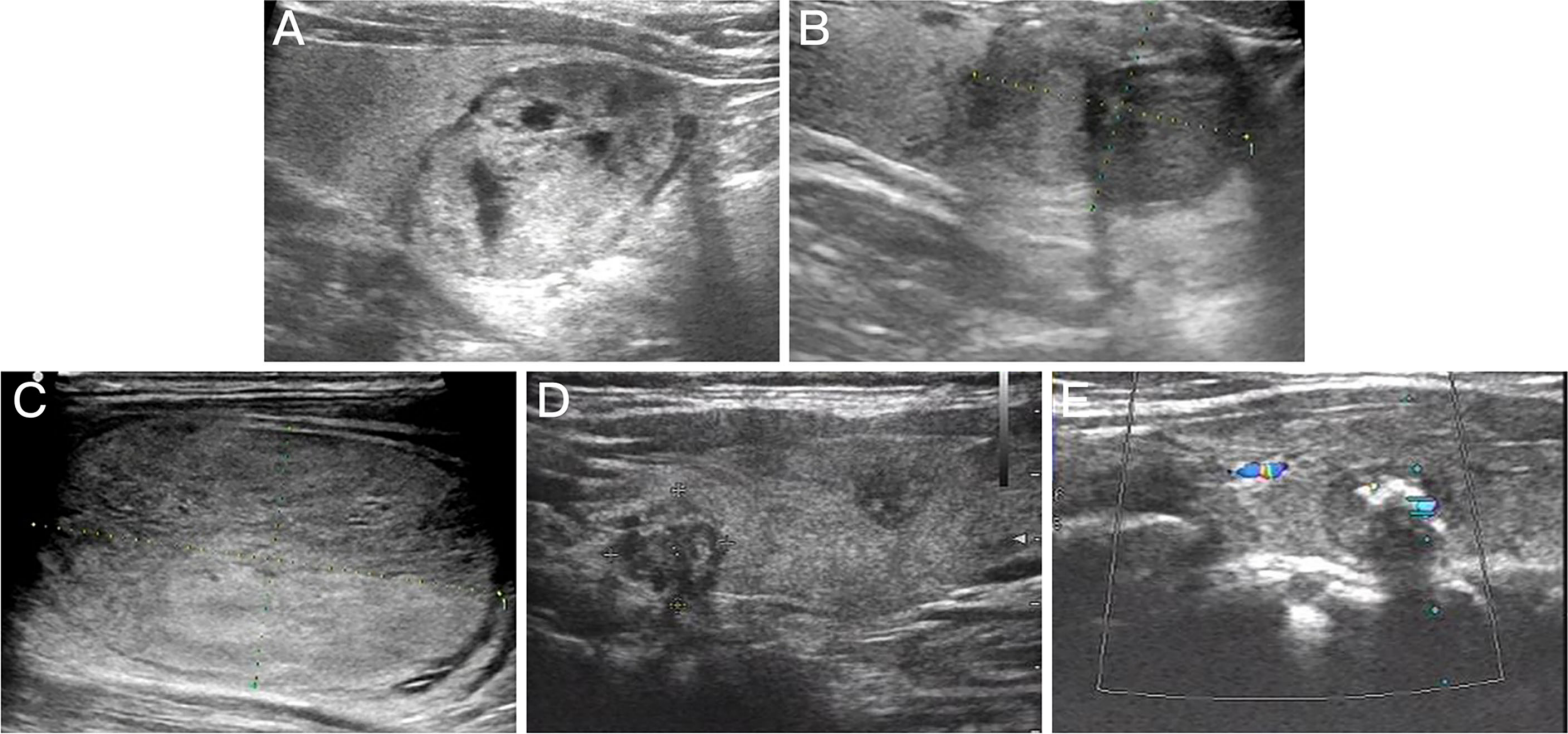
Preoperative ultrasound images for thyroid nodules with different pathologic types. **(A)** Follicular thyroid adenoma. **(B)** Follicular variant papillary thyroid carcinoma. **(C)** Follicular thyroid carcinoma. **(D)** Multifocal papillary thyroid carcinoma. **(E)** medullary carcinoma.

Preoperative ultrasound showed that the 47 Bethesda IV nodules in the MWA group were mainly characterized by isoechogenicity (95.74%) and no calcification (80.85%), and all thyroid nodules presented a regular margin with a wider-than-tall shape. Among the 84 patients with Bethesda IV nodules in the surgery group, 44 were diagnosed with benign thyroid nodules, 27 with PTC, 6 with FVPTC, 3 with FTC, and 1 with MTC by postoperative pathological examination. Four cases of borderline thyroid tumors were observed in the surgery group, including a hyalinizing trabecular tumor (HTT), a non-invasive follicular thyroid neoplasm with papillary-like nuclear features (NIFTP), a follicular tumor of uncertain malignant potential (FT-UMP), and a Hürthle cell carcinoma (HCC) (**Figure 2**). Preoperative ultrasound showed that the malignant thyroid nodules were more correlated with the characteristic of solid (86.11% *vs.* 72.72%), hypoechogenicity (55.56% *vs.* 13.63%), and irregular margin (47.22% *vs.* 13.63%) than benign lesions. Furthermore, there were significant differences in the composition, echogenicity, margin, and level of Thyroid Imaging Reporting and Data System of the American College of Radiology (ACR TI-RADS) of thyroid nodules that were postoperatively diagnosed as benign lesions, PTC, and FVPTC/FTC (all *p* < 0.05, **Table 2**).

**Table 2:** Preoperative ultrasound characteristics of patients with Bethesda category IV thyroid nodules (n = 132).

		Surgery group (n = 84)			
	MWA group (n = 47)				*p*
		**Benign lesions/FTA (n = 44)**	**PTC (n = 27)**	**FTC/FVPTC (n = 9)**	
Composition (n, %)					< 0.05
Solid	23 (47.95%)*	32 (72.72%)	24 (88.9%)	7 (77.78%)	
Cyst-solid	14 (29.79%)*	2 (4.54%)	3 (11.1%)	2 (22.22%)	
Cystic	10 (21.28%)*	10 (22.72%)	0	0	
Echogenicity (n, %)					< 0.05
Hypoechoic	2 (4.26%)*	6 (13.64%)	16 (59.26%)	4 (44.44%)	
Isoechoic/hyperechoic	45 (95.74%)*	38 (86.36%)	11 (40.74%)	5 (55.56%)	
Margin (n, %)					< 0.05
Regular	47 (100%)	38 (86.36%)	14 (51.85%)	5 (55.56%)	
Irregular	0	6 (13.64%)	13 (48.15%)	4 (44.44%)	
Calcification (n, %)					
Microcalcification	7 (14.89%)	6 (13.64%)	6 (22.22%)	1 (11.11%)	
Coarse/rim calcification	2 (4.26%)	9 (20.45%)	5 (18.52%)	3 (33.33%)	
Absent	38 (80.85%)	29 (65.9%)	16 (59.26%)	5 (55.56%)	
Shape					
Taller-than-wide	0	1 (2.27%)	4 (14.81%)	0	
Wider-than-tall	47 (100%)	43 (97.72%)	23 (85.19%)	9 (100%)	
ACR TI-RADS					< 0.05
TI-RADS 1	11 (23.40%)*	5 (11.36%)	2 (7.4%)	0	
TI-RADS 2	6 (12.77%)*	2 (4.54%)	0	1 (11.11%)	
TI-RADS 3	20 (42.56%)*	17 (38.63%)	6 (22.22%)	2 (22.22%)	
TI-RADS 4	10 (21.28%)*	17 (38.63%)	8 (29.63%)	4 (44.44%)	
TI-RADS 5	0	3 (6.8%)	11 (40.74%)	2 (22.22%)	

MWA, microwave ablation; FTA, follicular thyroid adenoma; PTC, papillary thyroid carcinoma; FTC, follicular thyroid carcinoma; FVPTC, follicular variant of papillary thyroid carcinoma; ACR TI-RADS, the Thyroid Imaging Reporting and Data System of American College of Radiology.

*p < 0.05 vs. MWA group.

### Follow-up of thyroid nodules after microwave ablation

**Figure 3:**
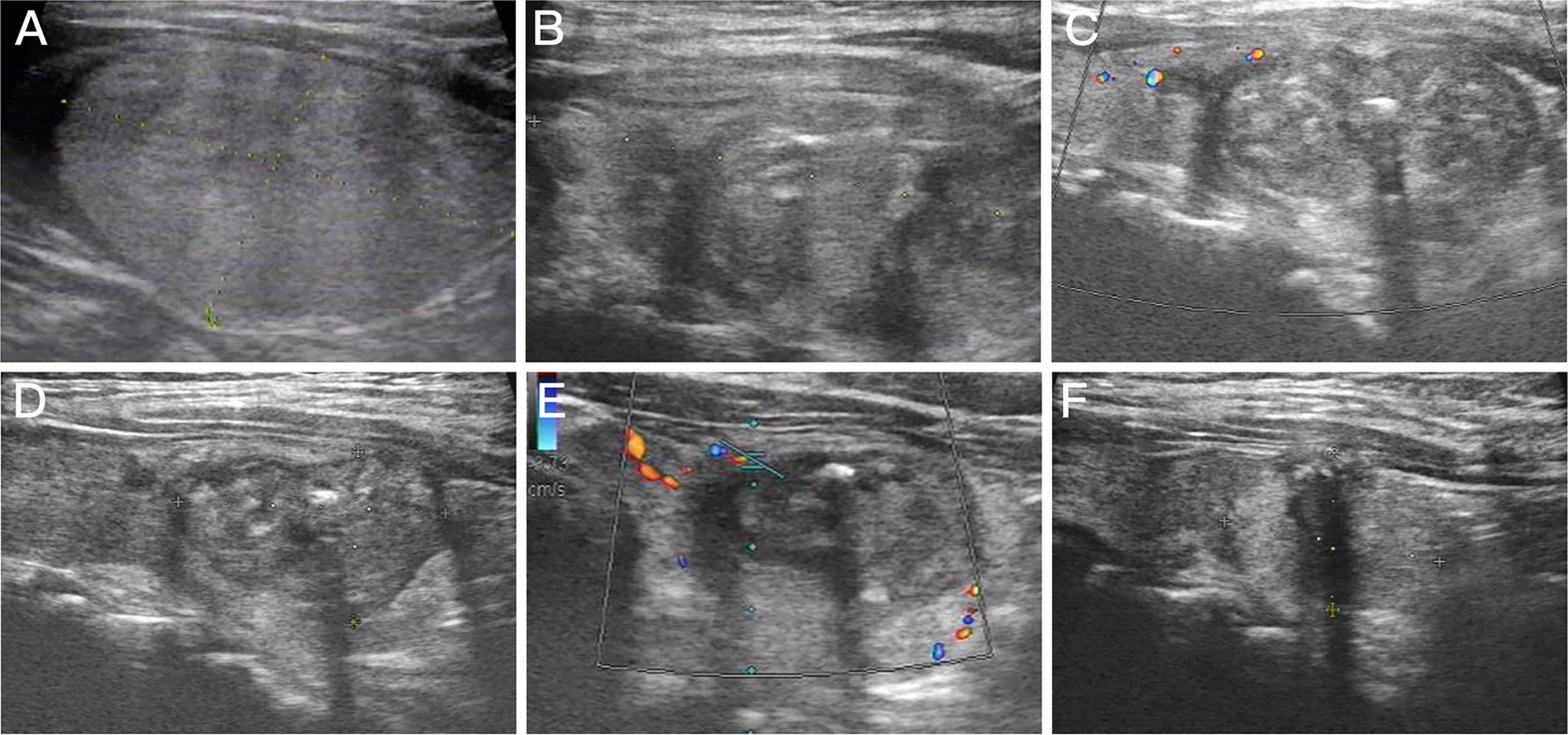
Imaging of a 63-year-old woman with Bethesda IV nodules who underwent microwave ablation (MWA). **(A)** An isoechoic solid thyroid nodule was detected in the left lobe with an initial volume of 14.29 ml. **(B)** Ultrasound examination showed enlarged ablation area (20.16 ml) immediately after MWA. **(C–F)** The volume of the ablation area was 12.39, 6.33, 3.13, and 2.04 ml at 1, 3, 6, and 12 months after MWA, respectively.

Patients in the MWA group were followed up for 10.05 ± 3.06 months, and the mean thyroid nodule volumes at 0, 1, 3, 6, and 12 months after MWA were 10.85 ± 10.18, 5.82 ± 4.55, 3.73 ± 3.38, 2.50 ± 2.63, and 1.42 ± 1.27 ml, respectively. The VRRs were 37.90% ± 22.54%, 58.59% ± 20.34%, 73.84% ± 17.24%, and 85.01% ± 10.86%, respectively. Recurrence and lymphatic or distant metastasis were not reported during the follow-up period. There were significant differences in the maximum diameter and volume of thyroid nodules between those measured at 3, 6, and 12 months postoperatively and those at baseline (all *p* < 0.05, **Figure 3**). Notably, the VRRs gradually increased with the prolongation of follow-up and were significantly higher at each time point of follow-up than that at baseline (all *p* < 0.001, **Table 3**).

**Table 3:** The maximum diameter, volume, and VRR changes of thyroid nodules after MWA.

		Maximum diameter		Volume		VRR (%)	
	n	Mean ± SD (cm)	*p*	Mean ± SD (ml)	*p*	Mean ± SD	*p*
Baseline	47	3.12 ± 0.86	—	7.47 ± 6.68	—	—	—
Immediately	47	3.28 ± 0.90	0.890	10.85 ± 10.18	0.060	—	–
1 month	46	2.80 ± 0.71	0.023*	5.82 ± 4.55	0.015*	37.90 ± 22.54	<0.001*
3 months	43	2.37 ± 0.62	<0.001*	3.73 ± 3.38	<0.001*	58.59 ± 20.34	<0.001*
6 months	43	2.01 ± 0.67	<0.001*	2.50 ± 2.63	<0.001*	73.84 ± 17.24	<0.001*
12 months	33	1.66 ± 0.61	<0.001*	1.42 ± 1.27	<0.001*	85.01 ± 10.86	<0.001*

MWA, microwave ablation; VRR, volume reduction rate.

*p < 0.05 vs. baseline.

### Postoperative complications after microwave ablation and surgery

Postoperative hoarseness, dysphagia, hematoma, and infection were not reported in the MWA group during the follow-up period. Only two cases in the MWA group developed postoperative pain, which spontaneously disappeared within 2 h. A total of 72 patients in the surgery group developed postoperative pain, which was relieved within 12 h. One patient in the surgery group suffered from glottic edema, incision swelling, and shortness of breath, which were alleviated after 1-day mechanical ventilation. One patient in the surgery group developed postoperative numbness of hands and feet, which spontaneously disappeared within 4 h. Serum calcium and parathyroid hormone of this case were in the normal ranges, and vocal cord paralysis was not examined by laryngoscopy. Another patient in the surgery group showed postoperative incision infection and hematoma without symptoms of fever. Laboratory examination data revealed a higher leukocyte count (13.76 × 10^9^/L) and neutrophil count (11.83 × 10^9^/L). The leukocyte count returned normal after a 3-day anti-infection treatment in the electronic intensive care unit (EICU). The incidence of postoperative complications was significantly higher in the surgery group than in the MWA group (*p* < 0.01, **Table 4**).

**Table 4:** Postoperative complications of patients with Bethesda IV nodules treated with MWA and surgery.

Complication	MWA group (n = 47)	Surgery group (n = 84)
Hoarseness (n, %)	0	0
Glottic edema (n, %)	0***	1 (1.19%)
Numbness of hands and feet (n, %)	0***	1 (1.19%)
Postoperative pain (n, %)	2 (4.26%)***	72 (85.71%)
Postoperative infection (n, %)	0***	1

Note. MWA, microwave ablation.

***p < 0.001 vs. surgery group.

### Treatment time and medical cost of microwave ablation and surgery

Compared with the surgery group, the MWA group presented significantly shorter treatment time (109.74 ± 42.42 *vs.* 4.03 ± 3.28 min) and length of stay (8.22 ± 2.24 *vs.* 5.15 ± 2.66 days), less cost (3,837.68 ± 2,385.11 *vs.* 2,617.42 ± 570.40 USD), and lower incision length (0 cm *vs.* 6.07 ± 1.93 cm), suggesting that MWA was more cost-effective than thyroid lobectomy/total thyroidectomy in the treatment of Bethesda IV nodules (*p* < 0.001, **Table 5**).

**Table 5:** Treatment time and medical cost of patients with Bethesda category IV thyroid nodules treated with MWA and surgery.

	MWA group (n = 47)	Surgery group (n = 84)
Total operation time (min)	4.03 ± 3.28***	109.74 ± 42.42
Hospitalization (day)	5.15 ± 2.66***	8.22 ± 2.24
Cost (USD)	2,617.42 ± 570.40***	3,837.68 ± 2,385.11
Incision length (cm)	0***	6.07 ± 1.93

MWA, microwave ablation.

***p < 0.001 vs. surgery group.

## Discussion

MWA is accomplished through oscillating charges from microwaves of 900–2,450 MHz to trigger violent movements of water molecules that increase the water temperature. Similarly, electromagnetic microwaves can induce cell death by heating (14). Ultrasound-guided MWA has been extensively applied in the minimally invasive treatment of liver, lung, and bone tumors (15–17). In recent years, the efficacy and safety of thermal ablation on benign thyroid nodules, PTC, metastatic lymph nodes of PTC, and papillary thyroid microcarcinoma (PTMC) have been verified (18–20). Our previous research has demonstrated that MWA, as a representative technique of thermal ablation, is an effective and safe strategy for low-risk PTMC (21). In the present study, MWA effectively reduced the volume of Bethesda IV thyroid nodules. There was no recurrence, lymph node metastasis, and distant metastasis during the follow-up period. Therefore, for patients who are not ineligible for or refuse surgery, MWA is a safe and effective option.

Bethesda IV nodules are cytologically defined as FN or SFN in the TBSRTC, involving FA, FTC, and FVPTC. Theoretically, preoperative cytological examination or even core needle biopsy (CNB) is unable to distinguish benign from malignant thyroid nodules. Recommended by the European Thyroid Association (ETA) and Cardiovascular and Interventional Radiological Society of Europe (CIRSE), surgery remains the standard management for thyroid nodules with Bethesda IV cytology, and ablation should be restricted to patients with follicular lesions who are at surgical risk and present favorable US features and negative molecular testing, as an alternative to clinical surveillance (19). In our study, preoperative ultrasound characteristics **Table 5** of 47 patients with Bethesda IV nodules in the MWA group included isoechoic nodules (95.7%), no calcification (80.85%), wider-than-tall with regular margin (100%), and TI-RADS 3 nodules (42.56%). Among the 36 malignant thyroid nodules diagnosed by postoperative pathology, there were 17 irregular nodules and 20 solid hypoechoic nodules (involving 16 PTC and 4 FVPTC cases). Significant differences were detected in the composition, echogenicity, margin, and ACR TI-RADS of benign lesions, PTC, and FTC. Taken together, surgery should be actively performed in patients with Bethesda IV nodules presenting suspected ultrasound characteristics like solid echotexture, hypoechogenicity, and irregular margins.

Anabella et al. (22) conducted a prospective analysis of 155 patients with single Bethesda IV nodules. Of these patients, 23 who refused surgery or were at a high risk of surgery were actively followed up, with a median duration of 3.5 years during which no evidence of tumor progression was observed. However, active surveillance may cause anxiety in patients, which probably impairs patients’ quality of life and compliance with follow-up (23). In the present study, 44/85 (51.76%) of Bethesda IV thyroid in the surgery group were diagnosed as benign lesions, involving 29 cases of FTA, nine nodular goiters, and six lymphocytic thyroiditis. Among the 37 Bethesda IV thyroid diagnosed as malignant lesions by postoperative pathology, there were 27 (72.97%) cases of PTC, 6 (16.21%) of FVPTC, 3 (4.47%) of FTC, and 1 (2.70%) of MTC. In particular, all three cases of FTC belonged to the subtype of minimally invasive follicular thyroid carcinoma and were treated with thyroid lobectomy. Lymph nodes and distant metastasis were not examined during the surgery, and vascular invasion was absent. Lee et al. (24) demonstrated that the occurrence of distant metastasis of minimally invasive follicular thyroid carcinoma is not influenced by the surgical extent (thyroid lobectomy *vs.* total thyroidectomy). It is reported that ablation of follicular neoplasms can delay surgery in case of malignancy, while it does not influence the subsequent treatment and postoperative pathology (25). Whether ablation accelerates the malignant progression from follicular neoplasms into follicular carcinoma and/or residual thyroid tumors remains unknown. In the present study, the ablation extent was 0.5–1 cm beyond the edge of the tumor to prevent tumor residues and recurrence. During the follow-up period, only one patient with a VRR of 65.79% was transferred to surgical treatment at 12 months and postoperatively diagnosed as FTA. Recurrence, lymph node metastasis, and distant metastasis were not reported in the remaining patients. Ha et al. (26) conducted a 5-year follow-up in 10 cases of small follicular neoplasms (<2.00 cm) treated with radiofrequency ablation, and they did not report recurrent or distant metastatic cases. Collectively, it is necessary to thoroughly assess benign and malignant nodules and general conditions of patients before MWA to prevent residual tumors and recurrence.

In our previous study, the median VRR of benign non-functioning thyroid nodules at 1 year after MWA reached 86.67% (27). A meta-analysis showed that the VRR of benign thyroid nodules treated with MWA was 63.00%–89.90% (28). According to the Proposal for Standardization of Terminology and Reporting Criteria for Image-Guided Thyroid Ablation proposed by the Italian Working Group on Minimally Invasive Treatments of the Thyroid, a 50% volumetric reduction at 1 year is considered a reasonable threshold for defining the technical success of ablation (29). In the present study, MWA was successful in all 47 patients with Bethesda IVnodules. The mean VRR was 85.01% ± 10.86% at 12 months, and the VRRs were more than 90.00% in 15 patients at the last time of follow-up. Dong et al. (30) reported that the VRR of benign thyroid nodules greater than 4 cm reaches 90% ± 5% at 12 months after MWA. In addition, MWA is evident in protecting thyroid function and alleviating clinical symptoms, like compression and lower cosmetic score than lobectomy. Our results showed that only two patients in the MWA group developed postoperative pain, which was reported in 72 patients of the surgery group. In addition, one case of postoperative glottic edema, incision swelling, and shortness of breath and one of postoperative infection and hematoma were reported in the surgery group. The successful ligation of blood vessels and immediate hemorrhage are critical to prevent hematoma during thyroid lobectomy. A patient in the surgery group developed numbness in his/her hands and feet. Cavicchi et al. (31) have shown that adequate exposure of the recurrent laryngeal nerve during surgery and its intermittent monitoring can effectively prevent surgical damage. During the MWA procedure, normal saline was continuously injected in the space between the thyroid capsule and trachea, esophagus, laryngeal nerve, and parathyroid gland to safeguard vital structures of the neck. The incidence of complications was significantly lower in the MWA group than in the surgery group. Moreover, the MWA group presented shorter treatment time and hospitalization time and less cost than the surgery group, suggesting that MWA is an effective, safe, and cost-effective option for patients with Bethesda IV nodules.

Several limitations in this study should be concerned. First, patients in the MWA group were only followed up for 12 months. The long-term efficacy of MWA on Bethesda IV nodules should be further observed in a longer follow-up. Second, patients in the surgery group were not followed up, and their postoperative quality of life, levothyroxine use, and adverse events were not analyzed. Third, it was a retrospective study with small sample size. A multi-center, large-scale prospective study with a longer follow-up period is needed in the future to validate our findings.

In conclusion, this study demonstrated that ultrasound-guided MWA can treat Bethesda IV nodules with a low incidence of complications. For patients ineligible for or refusing surgery, MWA is a safe and effective option. However, patients with suspected preoperative ultrasound characteristics like solid echotexture, hypoechogenicity, and irregular margins should be actively treated with surgery.

## Data availability statement

The raw data supporting the conclusions of this article will be made available by the authors, without undue reservation.

## Ethics statement

This study was reviewed and approved by ethics committee of the Jiangsu Province Academy of Traditional Chinese Medicine (MS2018074). The patients/participants provided their written informed consent to participate in this study.

## Author contributions

JY, YZ and SX developed the research questionnaire and wrote the protocol for this study. JY and YZ were responsible for data collection and analysis. XL, GC, XC and RL participated in the diagnosis. SX and JW were the operators for microwave ablation and surgery, respectively. YTZ and XH were responsible for the perioperative management. SX, FH and CL interpreted the results. JY and YZ wrote the article. SX and CL revised it critically for important intellectual content. All authors agreed to take responsibility for the integrity of the data and the accuracy of the data analysis. All authors contributed to the article and approved the submitted version.

## Funding

This study was funded by the Jiangsu Provincial Key R&D Programme (Social Development, BE2020726), Scientific Research Project of Jiangsu Health Committee (M2020102), Special Project on Diagnosis and Treatment of Key Clinical Diseases in Suzhou (LCZX201914), and Opening Project of National Clinical Research Base of Chinese Medicine (JD2019SZXYB13).

## Acknowledgments

We are grateful to the patients who participated in this study.

## Conflict of interest

The authors declare that the research was conducted in the absence of any commercial or financial relationships that could be construed as a potential conflict of interest.

## Publisher’s note

All claims expressed in this article are solely those of the authors and do not necessarily represent those of their affiliated organizations, or those of the publisher, the editors and the reviewers. Any product that may be evaluated in this article, or claim that may be made by its manufacturer, is not guaranteed or endorsed by the publisher.
